# Support Vector Machine with Ensemble Tree Kernel for Relation Extraction

**DOI:** 10.1155/2016/8495754

**Published:** 2016-03-22

**Authors:** Xiaoyong Liu, Hui Fu, Zhiguo Du

**Affiliations:** ^1^Department of Computer Science, Guangdong Polytechnic Normal University, Guangzhou, Guangdong 510665, China; ^2^College of Mathematics and Informatics, South China Agricultural University, Guangzhou, Guangdong 510642, China

## Abstract

Relation extraction is one of the important research topics in the field of information extraction research. To solve the problem of semantic variation in traditional semisupervised relation extraction algorithm, this paper proposes a novel semisupervised relation extraction algorithm based on ensemble learning (LXRE). The new algorithm mainly uses two kinds of support vector machine classifiers based on tree kernel for integration and integrates the strategy of constrained extension seed set. The new algorithm can weaken the inaccuracy of relation extraction, which is caused by the phenomenon of semantic variation. The numerical experimental research based on two benchmark data sets (PropBank and AIMed) shows that the LXRE algorithm proposed in the paper is superior to other two common relation extraction methods in four evaluation indexes (Precision, Recall, *F*-measure, and Accuracy). It indicates that the new algorithm has good relation extraction ability compared with others.

## 1. Introduction

Relation extraction task was formally put forward in MUC-7 by Message Understanding Conference (MUC) supported by Defense Advanced Research Projects committee (DARPA) in 1998. The development of relation extraction research was promoted by MUC and then ACE which replaced MUC later. In those two conferences, a variety of advanced information extraction methods were proposed. They were tested on the data platform provided by the conferences and discussed among the participants. The annual evaluation conference played a guiding and promoting role for the development of relation extraction.

As one of the important research tasks in the field of natural language processing research, interentity relation extraction has good application value in many fields. In the question answering system, relation extraction associates the related questions and answers automatically. In the retrieval system, it can realize the function of semantic retrieval. In the ontology learning process, it can find new interentity relations to enrich the ontology structure. In the annotation task of semantic web, it can automatically associate the knowledge unit.

At present, the studies on semisupervised relation extraction mainly focus on selection of initial seed set and pattern ordering issue. However, in semisupervised relation extraction, due to the small number of labeled corpora, to identify the relation of a large number of unlabeled corpora, it is necessary to extend the seed set in algorithm operation. If the extension process is not limited, it may cause a phenomenon of semantic variation due to the addition of misclassified new seeds into the seed set. In other words, with the addition of misclassified corpora into the seed set, when the seed set classifies other unlabeled corpora, errors expand continuously, which affects the classification accuracy. Thus, in order to solve the phenomenon of semantic variation, this paper proposes a SVM ensemble learning strategy based on subtree and subset tree kernel functions and introduces the semisupervised relation extraction method based on the constraint-mechanism seed set extension strategy.

## 2. Methodology and Materials

### 2.1. Methodology

#### 2.1.1. Related Methods

In the current research, relation extraction mainly includes three kinds of methods [1, 2]: supervised relation extraction, semisupervised relation extraction, and unsupervised relation extraction. Among them, the semisupervised learning method refers to a machine learning style in which the learning system learns automatically with several labeled samples and numerous unlabeled samples without human intervention. Semisupervised relation extraction method [3–9] usually gives a few corpus sets which have already been labeled the relational category marker as seed first and induces the pattern from those labeled corpora. Then, this pattern is applied to the sentence sets which have not been labeled the relational category to obtain new set pairs. Those new set pairs are added to seed set to continue the inducing pattern, so as to complete the relation extraction task on all unlabeled corpora. Greenwood and Stevenson [10] pointed out in the literature that, despite the good effects achieved, the semisupervised method for information extraction task is just limited in the pretreatment task, such as identification of event participants and senesce filtering without clearly revealing the degree of effect of the semisupervised method in relation extraction task. They put forward a relation extraction method based on semisupervised learning. The method uses the more complicated pattern and determines the similarity between relations based on kernel. Xu et al. develop a DARE system based on semisupervised relation extraction method [11–14]. In this system, the seed set (relation instance set) is specified by the user first to sum up the relation extraction pattern. Then, the pattern is applied to the corpora ready to be extracted to extract more relation instances until there are no more patterns and relation instances. In China, He et al. [15] put forward a named entity relation extraction method based on seed self-expansion, find out the named entity pairs, which appear in the same sentences, and the distance of them is under a certain value, and convert their contexts into vectors. A few named entity pair instances were selected and became initial relation seed set. The relation seed set is extended automatically in self-study process. Chen and Ji [16] proposed the graph-based semisupervised relation extraction method innovatively which uses label propagation algorithm to guide the computer to identify the interentity relations from unstructured texts automatically. This method first establishes the graph model of relation extraction with the graph strategy. In the graph model, all labeled and unlabeled examples are represented as the nodes and their distances as the weights of edges in the graph. Then, the task of relation extraction is converted into estimating a labeling function on this graph to satisfy the global consistency assumption. Cui et al. [17] propose extracting the interprotein relation with the SVM and active learning strategies. In addition, Wang et al. [18, 19] propose applying supervised and semisupervised learning mechanism into the extraction of protein relation. Chen et al. [20] combine the traditional characteristics of syntactic analysis with *n*-gram characteristics to make collaborative training, so as to improve the accuracy of relation extraction. Chen and Zhu [21] study the undefined-type semisupervised relation extraction issue based on the information of Wikipedia.

Converting relation extraction into classification task and adopting the classification algorithm in machine learning become an important research idea to solve the relation extraction task. In the research field of relation extraction, support vector machine (hereafter referred to as SVM) classification algorithm based on kernel function has received great attention in relation extraction research. And, semisupervised relation extraction algorithm has good practical value in solving the problem of a few labeled corpora and too many unlabeled corpora, in which bootstrapping-based semisupervised learning algorithm is an important method. Its algorithm process can be described in the following way [22]. Assuming two data sets of *D* (a data set with a small number of labeled) and *D*′ (a data set with a large number of unlabeled data) are given, it needs to label the category of samples in the unlabeled data set according to the information of the labeled data set. According to the training steps of Bootstrapping, the first is to train the classifier *C* on *D* well. Then, the categories of samples in *D*′ are judged with *C* and the results are incorporated into the original data set *D*. Next, *C* is trained and the rest data of *D*′ are classified again until all data in *D*′ are labeled. However, when adding new labeled samples to the data set *L*, the categories of samples added in *D* are often mistakenly labeled due to the wrong classification of samples by the trained classifier, thus leading to the labeling mistake when classifying other samples in *D*′ and then producing a phenomenon of semantic variation [23]. The influence of this phenomenon on the classification result gradually increases with the size of unlabeled samples.

The semisupervised relation extraction algorithm based on ensemble learning (LXRE algorithm) proposed in the paper mainly uses two SVM classification algorithms based on tree kernel function to train on the labeled data set and classify the data in the data set ready to be labeled. Only the samples with the consistent training results can be incorporated into the labeled data set. It uses the strategy of seed data set constraint expansion to increase the accuracy of relation extraction and weaken the inaccuracy of relation extraction caused by the semantic variation phenomenon of traditional semisupervised relation extraction algorithm.

#### 2.1.2. Tree Kernel

Collins and Duffy [24] first introduces tree kernel function into the field of natural language processing for grammar analysis and part-of-speech tagging. Later, some other scholars apply it to relation extraction and named entity recognition [25, 26]. Moschitti et al. [27] proposed several kernel functions to model parse tree properties in kernel-based machines, for example, perceptrons or support vector machines. In particular, they defined different kinds of tree kernels as general approaches to feature engineering in automated semantic role labeling. Plank and Moschitti [28] presented syntactic tree kernels enriched by lexical semantic similarity to tackle the portability of a relation extractor to different domains. Abad and Moschitti [29] introduced a simple method for weakly supervised relation extraction. It exploited syntactic information and lexical features by combining tree kernels with feature vectors and then applied SVM classier to handle overlapping relation problem. The main advantage of tree kernel is that it can produce a large number of grammatical features of the object and select some related features for specific tasks. Usually, tree kernel has two structures: one is known as subtree (ST) [30] and the other is known as subset tree (SST) [24]. Subtree contains a complete tree from one node and below. Subset tree does not include all leaf nodes under a certain node into the tree structure. It is more general than subtree.

**Figure 1:**
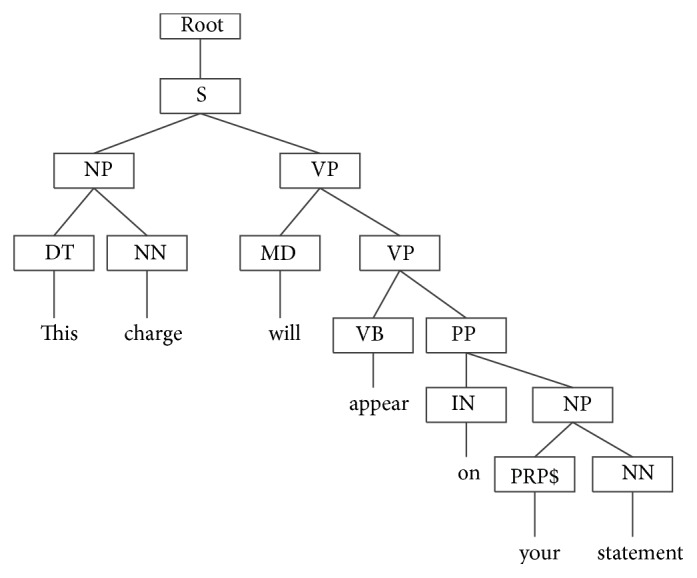
Syntax parsing tree of the example sentence.

**Figure 2:**
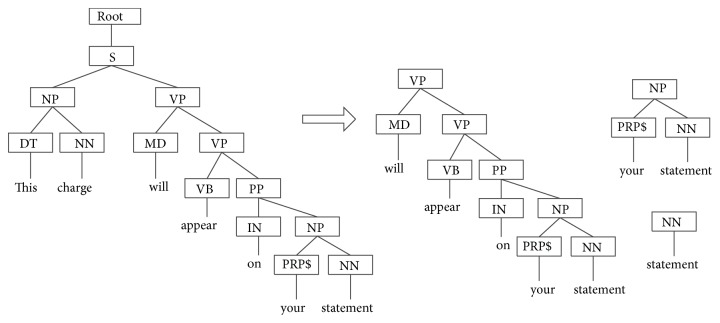
Partial subtree of the example sentence.

**Figure 3:**
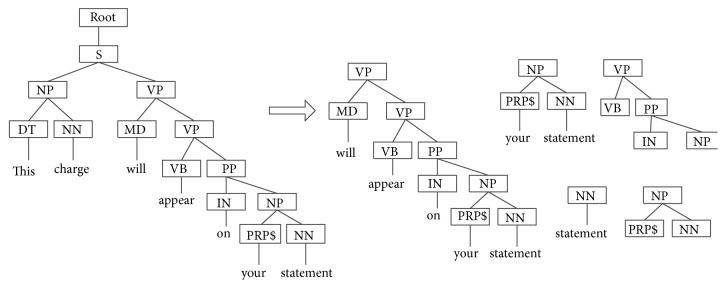
Partial subset tree of the example sentence.

The Stanford Parser is used to parse sentences from corpus in this paper. Stanford Parser is a statistical parser and is available for download as open source software at http://nlp.stanford.edu/software/lex-parser.shtml. For example, this charge will appear on your statement. Its syntax parsing tree is shown in Figure 1. Partial subtree and partial subset tree of the example are shown in Figures 2 and 3, respectively.

In the subtree of the example in Figure 2, all leaf nodes from one node and below are regarded as one part of subtree set. In the subset tree of the example in Figure 3, any subset tree does not necessarily include all the lead nodes below it.

The main concept of tree kernel calculation is to parse the similarity between trees through calculating the number of common subtrees between two parsing trees. For example, there are two parsing trees *T*_1_ and *T*_2_. Their similarity *K*_*c*_(*T*_1_, *T*_2_) can be calculated by the following formula [22]:

(1) $$\begin{array}{c} K_{c}\left(\;T_{1},T_{2}\;\right)=\overset{}{\underset{n_{1}\in N_{1},n_{2}\in N_{2}}{\sum }}\Delta \left(\;n_{1},n_{2}\;\right). \end{array}$$

Here, *N*_*i*_ is the node set of *T*_*i*_; Δ(*n*_1_, *n*_2_) is the number of common subtrees with *n*_1_ and *n*_2_ as the root.

#### 2.1.3. LXRE Algorithm

LXRE algorithm is expressed in the form of pseudocode as shown in Algorithm 1.

### 2.2. Materials

In order to verify the performance of the new algorithm, two benchmark test sets (Proposition Bank and AIMed) are used to evaluate the performance of the new algorithm. The original Proposition Bank project (PropBank), which is funded by ACE, created a corpus of text annotated with information about basic semantic propositions. The PropBank project has been extremely influential in recent research in natural language processing. AIMed corpus [31, 32] is developed by Bunescu et al. It mainly adopts 199 medical literature abstracts from PubMed to identify the interactive relations between proteins according to DIP. It is shown in Table 1 that those abstracts contain a total of 4026 sentences, including 951 with positive relation (i.e., there is a relation between proteins) and 3075 with negative relation (i.e., there is no relation between proteins).

## 3. Results and Discussion

### 3.1. Numerical Experiment in PropBank Corpus

To verify the performance of LXRE algorithm, the first test data set selected in the paper is PropBank [33] data set. Three algorithms ready to be tested are LXRE, N-LXRE, and N-RE, in which LXRE is the semisupervised relation extraction algorithm based on the seed set constraint extension mechanism proposed in the paper; N-LXRE is the semisupervised algorithm not using the constraint-mechanism seed set extension; N-RE is a supervised algorithm not extending the seed set completely.

#### 3.1.1. Algorithm Process

**Step 1 .**

Select several sentences from PropBank data set randomly as seed set for labeling and take other data sets as the text set ready to be labeled; make the syntactic parsing processing on each sentence to obtain the syntactic parsing tree set of all text sets; divide the labeled text texts into two parts of I1 and I2 equally.

**Step 2 .**

Train on I1 with svm based on ST or SST kernel function and on I2 with SVM based on ST or SST kernel function to obtain the classification model stProp.model or sstProp.model.

**Step 3 .**

Do the first labeling processing on unlabeled text set II with trained stProp.model or sstProp.model.

**Step 4 .**

Incorporate the texts in two models with the consistent classification into the initial labeled text set I to form candidate and then go to the next step.

**Step 5 .**

Calculate the similarity between the newly added texts and those in text set I through the syntax parsing tree; retain the newly added texts if the degree of similarity is not less than that between initial labeled texts; otherwise delete them and form new labeled text set.

**Step 6 .**

If all unlabeled texts II are labeled, quit the algorithm; otherwise, continue Steps 1 to 5.

#### 3.1.2. Experimental Results and Analysis

Three algorithms of LXRE, N-LXRE, and N-RE run independently for 10 times, respectively. The programs of those algorithms are written by Matlab R2014b and run on a computer with 2.0 GHz CPU, 2 GB DDR RAM.  Table 2 lists the appropriate values of these parameters in three algorithms. The results of 10 times of running are shown in Tables 2, 3, and 4 according to different data set capacities. The test indexes selected in the paper are Precision, Recall, *F*-measure, and Accuracy.

**Figure 4:**
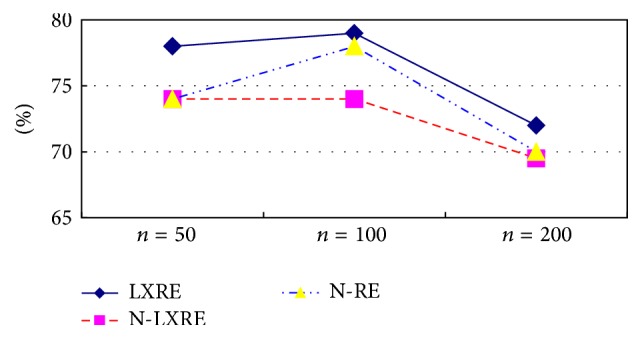
Experimental result comparison of three algorithms based on ST kernel function.

**Figure 5:**
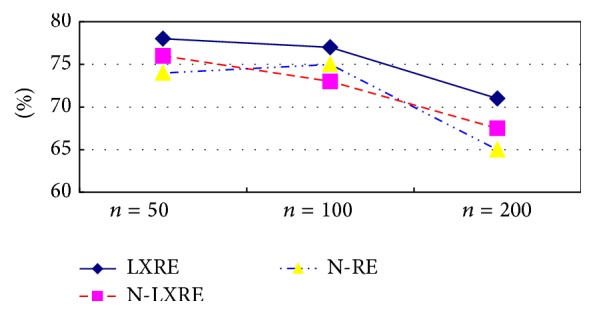
Experimental result comparison of three algorithms based on SST kernel function.

Figures 4 and 5 show the experimental result comparison of three algorithms on three data sets with different capacities based on ST and SST kernel functions, respectively. It can be intuitively found from the experimental result of the extraction algorithm based on ST kernel function in Figure 4 that LXRE has the best performance, followed by N-LXRE and N-RE. The experimental result of the extraction algorithm based on SST kernel function in Figure 5 also reflects that the test result of LXRE is the best on the data set. Except the poor performance on the data set with the capacity of 100, N-LXRE has better performance on other two data sets than N-RE.

Two conclusions can be drawn from the analysis of Figures 4 and 5:

1. The semisupervised extraction algorithm based on constraint-mechanism seed set extension can obtain stable and good effect in performance.
2. Since the algorithm without constraint-mechanism seed set extension has no constraint on adding into the seed set, some sentences which do not conform to the original seed set pattern (they may be classified mistakenly) are incorporated into the seed set and trained on it, which may reduce the performance when testing the unlabeled sentences. As a result, the performance of the seed set expansion algorithm without constraint mechanism may be poorer than that of N-RE algorithm on the data set with the capacity of 100. It also shows that the phenomenon of semantic variation of N-LXRE has certain effect on the result.

**Figure 6:**
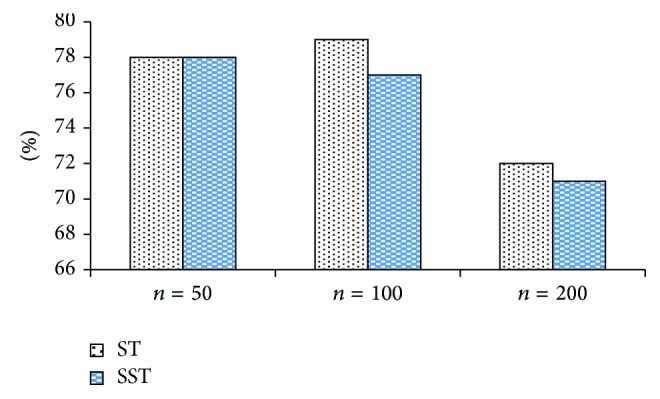
Classification accuracy comparison of LXRE based on ST and SST.

**Figure 7:**
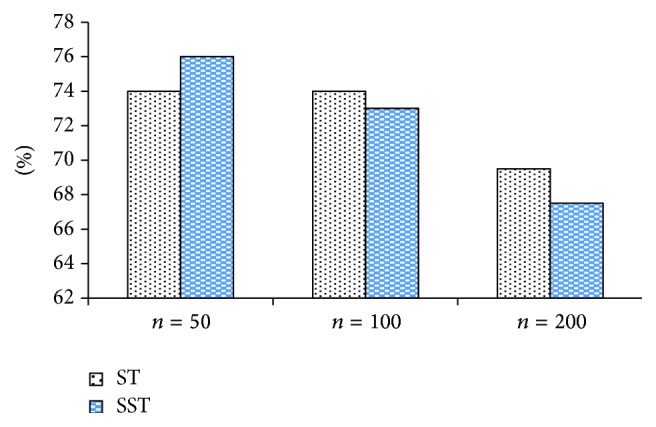
Classification accuracy comparison of N-LXRE based on ST and SST.

**Figure 8:**
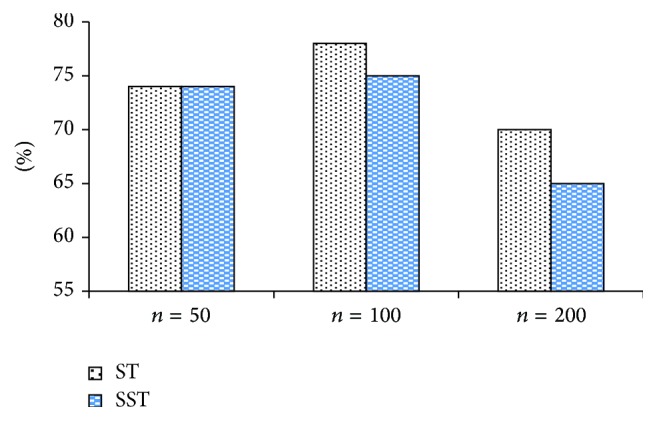
Classification accuracy comparison of N-RE based on ST and SST.

Figures 6, 7, and 8 make horizontal comparison of the performances of different algorithms on three data sets based on ST and SST tree kernel functions, respectively.

According to Figures 6, 7, and 8, the test on the data set with the capacity of 50 shows that the kernel function algorithm based on SST structure can obtain the similar and even better performance than that based on ST structure. With the increase of capacity of the data set, three algorithms based on ST kernel function are better than those based on SST structure. This result shows that the kernel function algorithm based on ST structure has good performance on the data set with large capacity while that based on SST structure has good result on the data set with small capacity. This conclusion indicates that the size of data set will directly affect the performance of algorithms based on ST and SST structures. In the actual application, it is generally feasible to select the tree kernel function algorithm according to the size of the data base.

To further analyze the performance advantage of LXRE algorithm compared with N-LXRE and N-RE, here, the percentages increased by comparing the best and poorest results between LXRE and other two algorithms in experiment are shown in Table 5.

It can be seen from Table 4 that, compared with other two algorithms, the accuracy index of LXRE has certain improvement on three data sets with the increasing range of (1.28%, 9.23%). Among them, LXRE algorithm based on SST has obvious improvement effect with the increase of capacity of the data set. From *n* = 50 to *n* = 200, its percentage increases by nearly 3% compared with that of the best result and by nearly 4% compared with that of the poorest result. At the same time, it can be found that, with the increase of capacity of the data set, the performance improvement effect of the algorithm is more obvious.

Thus, on the data sets of this experiment, the semisupervised learning method (LXRE) using the constrained extension seed set proposed in the paper is superior to other two methods in four indexes (Precision, Recall, *F*-measure, and Accuracy). N-LXRE semisupervised learning method has better effect than N-RE method which does not expand the seed set completely.

### 3.2. Numerical Experiment in AIMed Corpus

To further verify the performance of LXRE algorithm, the second common corpus AIMed is adopted to evaluate the performance of the algorithm. In the experiment, 1000 samples are selected randomly from AIMed corpus to form a test set, including 500 samples with positive relation and 500 with negative relation. Three algorithms' result comparison on AIMed is showed in Table 6. The experimental result shows that, except the consistency in Recall between Mitsumori's algorithm and LXRE, the values of *F*-measure and Precision of LXRE are better than those of other two algorithms, in which *F*-measure which reflects the comprehensive effect of the algorithm increases by 44.79% and 1.38% compared with that of other two algorithms, respectively.

**Figure 9:**
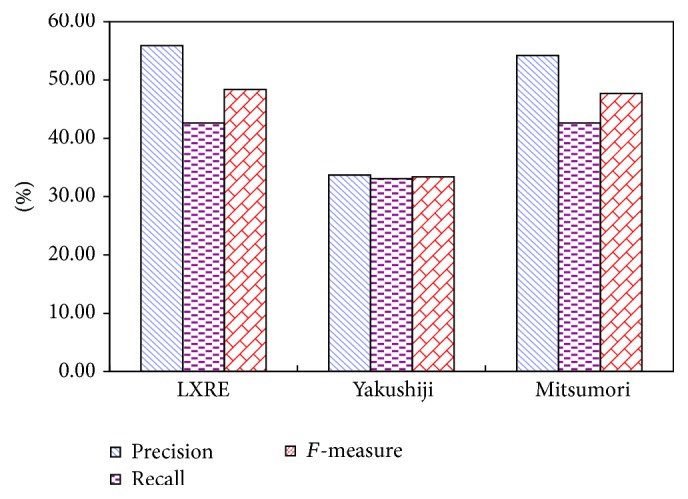
Comparison diagram of three algorithms (1).

**Figure 10:**
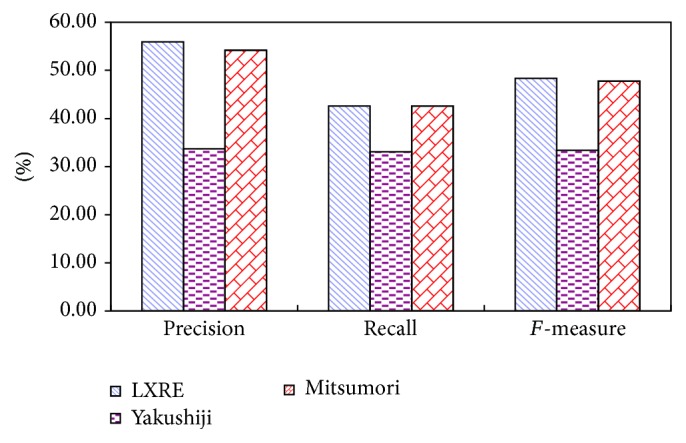
Comparison diagram of three algorithms (2).

In the algorithm proposed by Erkan et al. [36], since the *F*-measure result on the data set containing 1000 sample points is showed by graph, it is impossible to make the direct comparison. However, it can be found from its *F*-measure graph that on AIMed data set, when the capacity is 1000, the best *F*-measure values obtained by different algorithms are close to 50%, which is close to the result of this paper (Figures 9 and 10).

## 4. Conclusion

This paper carries out the semisupervised relation extraction with the SVM classification algorithm based on tree kernel function. To solve the common problems of the existing relation extraction method in relation extraction, it integrates two relation extraction algorithms based on subtree and subset tree kernel functions, respectively, and makes use of the seed set expansion strategy with the initial classification result of the algorithm to complete the new algorithm of relation extraction task through iteration. LXRE algorithm proposed in the paper shows good performance on three data sets from PropBank with different capacities. At the same time, the test result of new algorithm on AIMed, the interactive relation data set of medical protein, also shows that, compared with other three known relation extraction algorithms, LXRE has good relation identification performance. Relation identification of complex structure has been always a difficulty in relation extraction. Thus, the new algorithm proposed in the paper will be used in the research of this issue in the future.

## Figures and Tables

**Algorithm 1:**
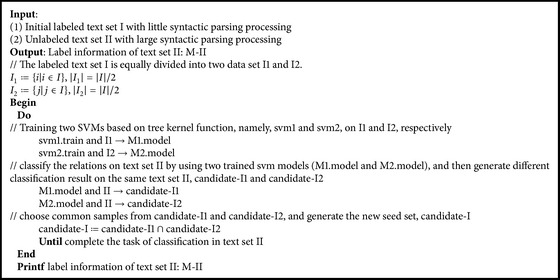


**Table 1:** Data set of interactive relations between proteins in AIMed.

Data set	Sentences	Positive	Negative
AIMed	4026	951	3075

**Table 2:** Algorithm comparison results on the data set with the capacity of 50.

	Y	*N*	Total	Precision	Recall	*F*-measure	Accuracy
LXRE							
ST	39	11	50	75.00%	84%	79.25%	78%
SST	39	11	50	81.82%	72%	76.60%	78%
N-LXRE							
ST	37	13	50	68.75%	88%	77.19%	74%
SST	38	12	50	76.00%	76%	76.00%	76%
N-RE							
ST	37	13	50	68.75%	88%	77.19%	74%
SST	37	13	50	87.50%	56%	68.29%	74%

**Table 3:** Algorithm comparison results on the data set with the capacity of 100.

	Y	*N*	Total	Precision	Recall	*F*-measure	Accuracy
LXRE							
ST	79	21	100	82.05%	69.57%	75.30%	79%
SST	77	23	100	87.10%	58.70%	70.13%	77%
N-LXRE							
ST	74	26	100	83.33%	54.35%	65.79%	74%
SST	73	27	100	82.76%	52.10%	63.94%	73%
N-RE							
ST	78	22	100	77.27%	73.91%	75.55%	78%
SST	75	25	100	88.89%	52.17%	65.75%	75%

**Table 4:** Algorithm comparison results on the data set with the capacity of 200.

	Y	*N*	Total	Precision	Recall	*F*-measure	Accuracy
LXRE							
ST	144	56	200	76.83%	63.00%	69.23%	72%
SST	142	58	200	80.00%	56.00%	65.88%	71%
N-LXRE							
ST	139	61	200	80.00%	52.00%	63.03%	69.5%
SST	135	65	200	79.66%	47.00%	59.12%	67.5%
N-RE							
ST	140	60	200	72.22%	65.00%	68.42%	70%
SST	130	70	200	81.25%	39.00%	52.70%	65%

**Table 5:** Percentages increased by comparing the best and worst results between LXRE and other two algorithms.

	*n* = 50		*n* = 100		*n* = 200	
	Best	Worst	Best	Worst	Best	Worst
ST	5.41%	5.41%	1.28%	6.76%	2.86%	3.60%
SST	2.63%	5.41%	2.67%	5.48%	5.19%	9.23%

**Table 6:** Algorithm result comparison on AIMed.

	Precision	Recall	*F*-measure
LXRE	55.91%	42.60%	48.36%
Yakushiji [34]	33.70%	33.10%	33.40%
Mitsumori [35]	54.20%	42.60%	47.70%
